# SOX4 induces cisplatin resistance in cervical cancer cells by inhibiting aerobic glycolysis

**DOI:** 10.1038/s41420-026-02954-x

**Published:** 2026-03-14

**Authors:** Ruili Sun, Haofei Gong, Ran Zhao, Huiying Lv, Huijie Yang, Xiaofei Zhu, Xinlai Qian, Jian Li, Qiongzi Wang

**Affiliations:** 1 Henan International Joint Laboratory of Immunology and Model Animals, School of Medical Technology, Xinxiang Medical University, Xinxiang, China; 2 Henan Collaborative Innovation Center of Molecular Diagnosis and Laboratory Medicine, School of Laboratory Medicine, Xinxiang Medical University, Xinxiang, China; 3 Department of Pathology, The Third Affiliated Hospital of Xinxiang Medical University, Xinxiang, China; 4 Xinxiang Key Laboratory of Tumor Microenvironment and Immunotherapy, Xinxiang Medical University, Xinxiang, China; 5 Department of Clinical Laboratory, The Third Affiliated Hospital of Xinxiang Medical University, Xinxiang, China; 6 Department of Pathology, The First Affiliated Hospital of Xinxiang Medical University, Xinxiang, China

**Keywords:** Cervical cancer, Cancer metabolism

## Abstract

Cisplatin resistance remains a major cause of chemotherapy failure in cervical cancer. Although our previous work identified that SOX4 promotes cisplatin resistance in cervical cancer cells, the underlying mechanism has not been fully elucidated. Here, we demonstrated that SOX4 not only induces resistance to cisplatin but also to oxaliplatin and carboplatin, suggesting its potential role as a multidrug resistance gene. Overexpression of SOX4 markedly suppressed glycolytic activity in cervical cancer cells and induced cisplatin resistance by inhibiting both the intrinsic and extrinsic apoptotic pathways. Rescue and neutralization experiments further indicated that SOX4 upregulates SIRT1, which subsequently represses the expression of GLUT1 on the cell membrane. This suppression leads to diminished cellular glucose uptake, resulting in decreased glycolysis and overall metabolic activity. Given that cisplatin preferentially targets highly proliferating cells, SOX4-driven metabolic deceleration enables cervical cancer cells to evade cisplatin-mediated cytotoxicity. Together, these findings demonstrate that SOX4 enhances cisplatin resistance in cervical cancer through SIRT1-upregulated suppression of glycolysis.

## Introduction

Cervical cancer remains one of the most common malignant tumors in gynecology. In recent years, factors such as environmental pollution and unhealthy lifestyle habits have contributed to a rising incidence and a trend toward younger onset of the disease [1]. According to the 2024 global cancer statistics, 660,000 new cervical cancer cases and 350,000 related deaths were reported in 2022 [2]. Persistent infection with high-risk human papillomavirus (HPV) is well established as the primary cause of cervical cancer. Although HPV vaccination has demonstrated efficacy in primary prevention, its protection is limited to uninfected individuals and does not cover all oncogenic HPV types [3]. Therefore, further research into improved strategies for the prevention and treatment of cervical cancer remains imperative.

SOX4 (SRY-related HMG-box 4) is a crucial transcription factor that plays an important role in regulating cellular processes such as proliferation, differentiation, migration, and apoptosis. In recent years, the function of SOX4 in cancer has garnered widespread attention. Numerous studies have demonstrated that SOX4 is overexpressed in multiple cancer types, including breast, lung, and prostate cancer, where it is closely associated with proliferation, epithelial-mesenchymal transition (EMT), tumor invasion, and metastasis [4]. SOX4 promotes tumor progression by regulating signaling pathways, including Wnt/β-catenin [5], PI3K/AKT [6], and TGF-β [7]. In cervical cancer, SOX4 forms regulatory axes with various non-coding RNAs, such as lncRNA and circRNA, to modulate the expression of key microRNAs. This, in turn, affects signaling pathways like Wnt/β-catenin and promotes tumor proliferation, invasion, and metastasis, or in some cases, inhibits the malignant progression of tumors [5, 8–13]. Additionally, high SOX4 expression is frequently correlated with poor prognosis, establishing it as an emerging focus in tumor diagnosis and therapeutic research [14]. While the involvement of SOX4 in EMT and metastasis has been extensively documented, its role in tumor metabolic reprogramming and chemoresistance remains poorly understood. Our research suggests that SOX4 may regulate metabolic processes such as glycolysis through modulation of metabolism-related genes, thereby contributing to chemoresistance in cervical cancer cells. This study focuses on elucidating the role of SOX4 in regulating glucose metabolism and chemoresistance, with the aim of identifying novel therapeutic targets for overcoming drug resistance.

In normal cells, the primary energy source is oxidative phosphorylation within mitochondria, whereas tumor cells frequently rely on aerobic glycolysis to meet their metabolic demands. This phenomenon, known as the “Warburg effect,” represents a metabolic reprogramming that occurs in cancer cells and is one of the hallmark traits of tumors [15, 16]. To accommodate this metabolic shift, tumor cells typically upregulate the expression of glucose transporters (GLUTs) [17] to enhance glucose uptake. Furthermore, by increasing the expression and activity of key rate-limiting enzymes, such as hexokinase (HK), phosphofructokinase (PFK), and pyruvate kinase (PK) [18], tumor cells markedly improve glycolytic efficiency, rapidly converting nutrients into energy and metabolic precursors to sustain their rapid growth and proliferation. Consequently, rapidly proliferating cancer cells become the primary targets of conventional chemotherapeutic agents like cisplatin. Glycolytic metabolic reprogramming is not only closely associated with tumor initiation and progression but also plays a significant role in chemoresistance [19]. Enhanced activity of glycolytic rate-limiting enzymes significantly increases glycolysis levels and lactate accumulation, providing the metabolic foundation for resistance development [20]. Studies have indicated that overexpression of HK2, PFK, and PKM2 in various solid tumors, including ovarian and lung cancers, is strongly associated with resistance to platinum-based drugs, 5-fluorouracil, doxorubicin, and other chemotherapy agents [21, 22]. Notably, recent research has uncovered a “secondary” reprogramming event: during the formation of cisplatin-resistant cells, the energy metabolism shifts from aerobic glycolysis back to oxidative phosphorylation [23]. Evidence suggests that, compared to cisplatin-sensitive cells, resistant cells downregulate GLUT1 expression via SIRT1, resulting in reduced glucose uptake and consumption [24].

SIRT1 is an NAD⁺-dependent deacetylase that plays a crucial role in tumor metabolic reprogramming and chemoresistance through the regulation of key metabolic molecules and signaling pathways. SIRT1 enhances the stability and activity of HIF-1α and MYC through deacetylation, thereby upregulating the expression of glycolysis-related genes such as GLUT1, HK2, and LDHA. This increases glucose uptake and lactate production in tumor cells, promoting the Warburg effect [25, 26]. Moreover, SIRT1 is closely associated with chemoresistance. On one hand, the Warburg effect reduces the efficacy of chemotherapeutic drugs that target rapidly proliferating cells. On the other hand, SIRT1 contributes to chemoresistance by deacetylating and inhibiting apoptosis-related proteins, such as p53 and Bax, thereby reducing chemotherapy-induced apoptosis and further enhancing tumor cell resistance [27]. These findings highlight the dual role of SIRT1 in regulating both tumor metabolism and drug resistance, underscoring its potential as a therapeutic target in cancer treatment.

This study aims to investigate the molecular mechanism through which SOX4 regulates SIRT1 to suppress aerobic glycolysis and induce chemoresistance in cervical cancer cells. By elucidating the role of this SOX4-SIRT1 pathway in metabolic reprogramming, we seek to identify novel therapeutic targets for overcoming drug resistance and improving cervical cancer treatment outcomes.

## Results

### SOX4 induces cisplatin resistance in cervical cancer cells

**Fig. 1:**
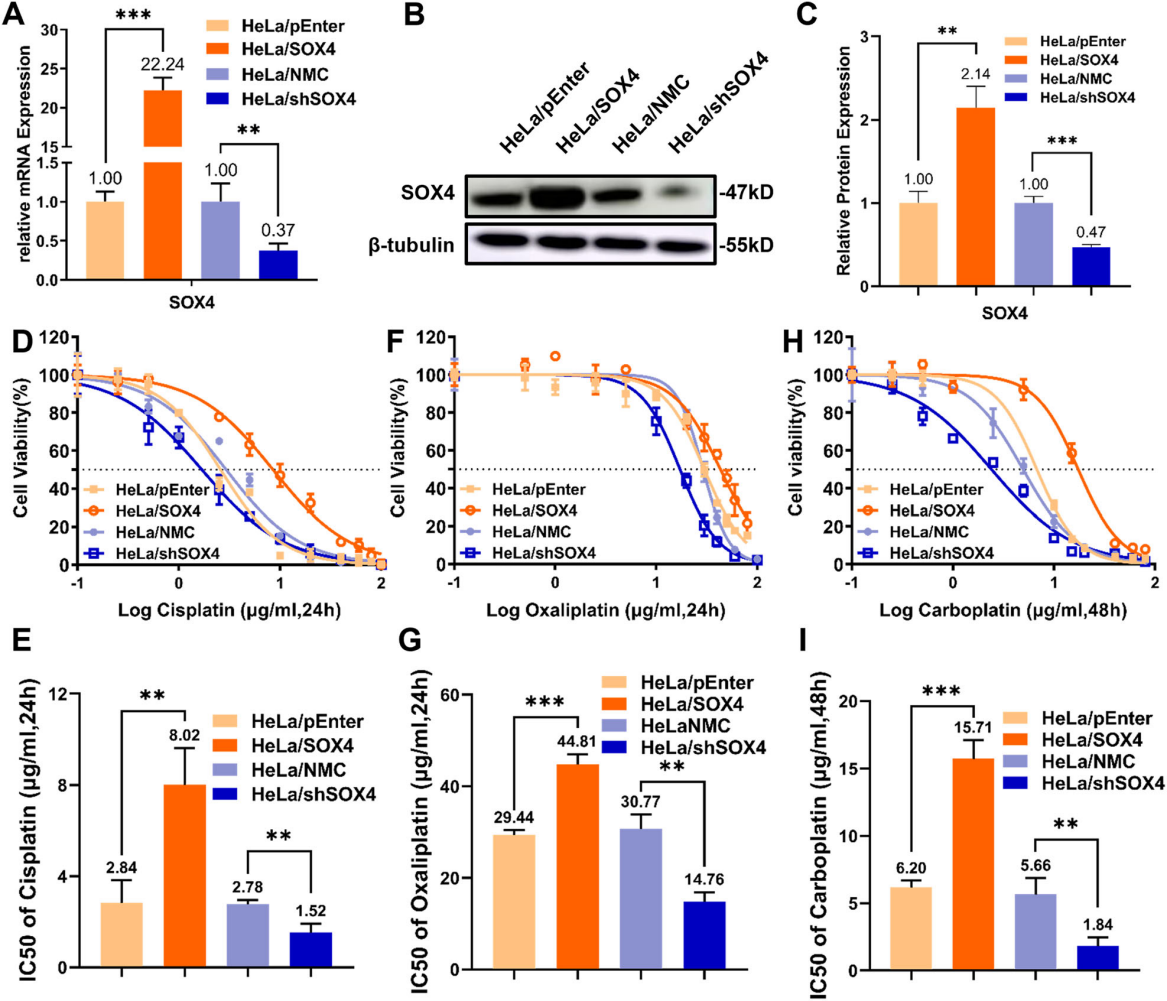
SOX4 induces resistance to platinum-based drugs in cervical cancer cells. **A**–**C** HeLa cell lines with SOX4 overexpression and knockdown were successfully established. Transfection efficiency was assessed by qPCR (**A**) and Western blot (**B**), with (**C**) showing the quantification of (**B**). Chemoresistance was evaluated by determining the IC50 values of cisplatin (**D**, **E**), oxaliplatin (**F**, **G**), and carboplatin (**H**, **I**) in SOX4-modified HeLa cells using the CCK-8 assay. ^*^p < 0.05, ^**^p < 0.01, ^***^p < 0.001.

To investigate the role of SOX4 in cervical cancer, we established stable cervical cancer cell lines with either SOX4 overexpression or knockdown. qPCR results showed that SOX4 mRNA levels in HeLa cells overexpressing SOX4 (HeLa/SOX4) were 22.24 times higher than those in the control group (HeLa/pEnter). In contrast, SOX4 expression in the knockdown group (HeLa/shSOX4) was reduced by 63% compared to the control cells (HeLa/NMC) (Fig. 1A). Western blot analysis further confirmed that SOX4 protein expression in HeLa/SOX4 cells was 2.14 times higher than in HeLa/pEnter cells, while its level in HeLa/shSOX4 cells was only 47% of that in HeLa/NMC cells (Fig. 1B, C). These results confirm the successful establishment of SOX4-modulated cell lines, which were thereafter utilized in subsequent experiments.

To evaluate the effect of SOX4 on chemosensitivity, SOX4-modulated cervical cancer cells were treated with commonly used platinum-based chemotherapeutic agents, including cisplatin, oxaliplatin, and carboplatin. The results showed that the IC50 values in HeLa/SOX4 cells were 8.02 μg/ml for cisplatin, 44.81 μg/ml for oxaliplatin, and 15.71 μg/ml for carboplatin. In contrast, the IC50 values in the control HeLa/pEnter cells were 2.84 μg/ml, 29.44 μg/ml, and 6.20 μg/ml, respectively, indicating that SOX4 overexpression increased the IC50 values by 2.8-fold, 1.5-fold, and 2.5-fold for each drug. Conversely, HeLa/shSOX4 cells exhibited significantly reduced IC50 values of 1.52 μg/ml(cisplatin), 14.76 μg/ml(oxaliplatin), and 1.84 μg/ml(carboplatin). These values were notably lower than those in the control HeLa/NMC cells, which were 2.78 μg/ml, 30.77 μg/ml, and 5.66 μg/ml, corresponding to reductions to 54.7%, 48.0%, and 32.5% of the control values (Fig. 1D–I).

These results indicate that SOX4 overexpression confers robust resistance to platinum-based agents in cervical cancer cells, while its knockdown restores cellular sensitivity. Collectively, these findings support the potential role of SOX4 as a multidrug resistance gene in cervical cancer.

### SOX4 suppresses cisplatin-induced apoptosis in cervical cancer cells via intrinsic and extrinsic apoptosis pathways

**Fig. 2:**
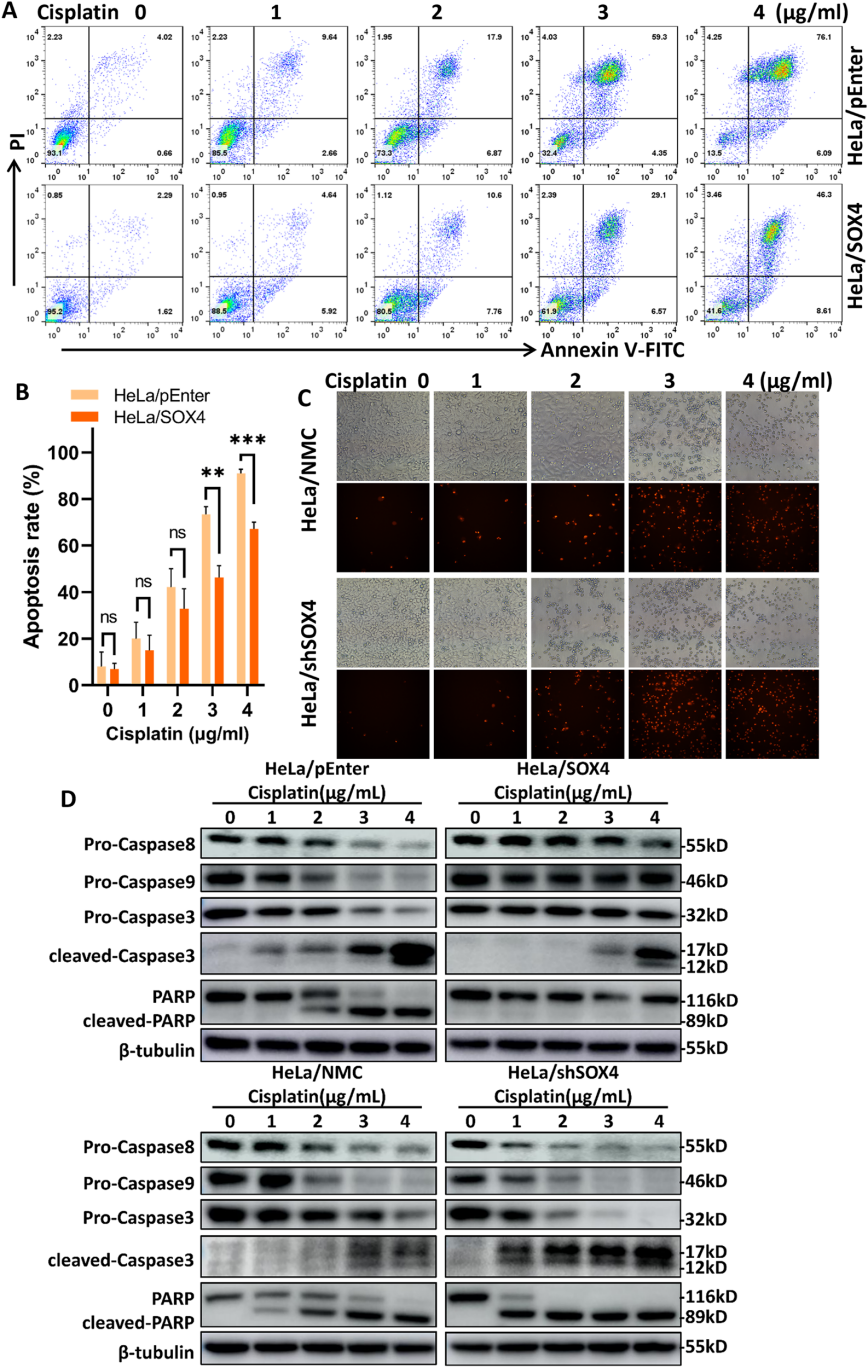
SOX4 suppresses cisplatin-induced apoptosis via intrinsic and extrinsic pathways. HeLa cells overexpressing or with knocked-down SOX4, along with their respective control cells, were exposed to varying concentrations of cisplatin (0, 1, 2, 3, 4 μg/mL) for 24 h. **A** Annexin V-FITC/PI dual staining followed by flow cytometry to evaluate cell apoptosis in SOX4-overexpression cell lines. **B** Quantification of apoptosis rates from (**A**) (*n* = 3). **C** PI single staining in SOX4-knockdown cell lines, with apoptosis observed under a fluorescence microscope. **D** Western blot analysis of apoptotic protein expression, including Caspase-3, Caspase-8, Caspase-9, and PARP. ^*^p < 0.05, ^**^p < 0.01, ^***^p < 0.001.

Cisplatin is a frontline chemotherapy drug for cervical cancer and was selected as a representative drug for subsequent experiments. To investigate the effect of SOX4, HeLa cells overexpressing SOX4 and their control counterparts were treated with increasing concentrations of cisplatin. Flow cytometry analysis revealed a marked difference in apoptosis rates between HeLa/pEnter and HeLa/SOX4 cells at 3 μg/mL cisplatin, with values of 73.42% and 46.34%, respectively, reflecting a 36.9% reduction. At 4 μg/mL, the apoptosis rates were 90.93% and 67.09%, corresponding to a 26.2% decrease (Fig. 2A, B).

Due to the presence of GFP fluorescence in HeLa/shSOX4 cells, Annexin V-FITC/PI dual staining was unsuitable for flow cytometry. Therefore, apoptosis was assessed using PI single staining followed by fluorescence microscopy. The results indicated that HeLa/shSOX4 cells exhibited enhanced apoptosis rates upon cisplatin treatment. Notably, at 3 μg/mL, the majority of HeLa/NMC cells remained viable whereas HeLa/shSOX4 cells showed near-complete apoptosis (Fig. 2C). These findings suggest that SOX4 inhibits apoptosis in HeLa cells.

Previous studies indicate that cisplatin induces apoptosis through both the extrinsic pathway (mediated by Caspase-8) and the intrinsic pathway (via Caspase-9), ultimately leading to Caspase-3 and PARP activation [28]. To determine how SOX4 modulates cisplatin-induced apoptosis, we conducted Western blot analyzes. The results showed that in HeLa/pEnter and HeLa/NMC cells, Caspase-3, -8, -9, and PARP were activated at cisplatin concentrations of 2–3 μg/mL. In contrast, HeLa/SOX4 cells required a higher concentration (≥4 μg/mL) to achieve similar activation. Conversely, in HeLa/shSOX4 cells, these apoptotic proteins were activated at only 1 μg/mL cisplatin, resulting in pronounced apoptosis (Fig. 2D).

These results demonstrate that SOX4 suppresses cisplatin-induced apoptosis in cervical cancer cells by inhibiting both intrinsic and extrinsic apoptotic pathways.

### SOX4 induces chemotherapy resistance in cervical cancer cells in vivo

**Fig. 3:**
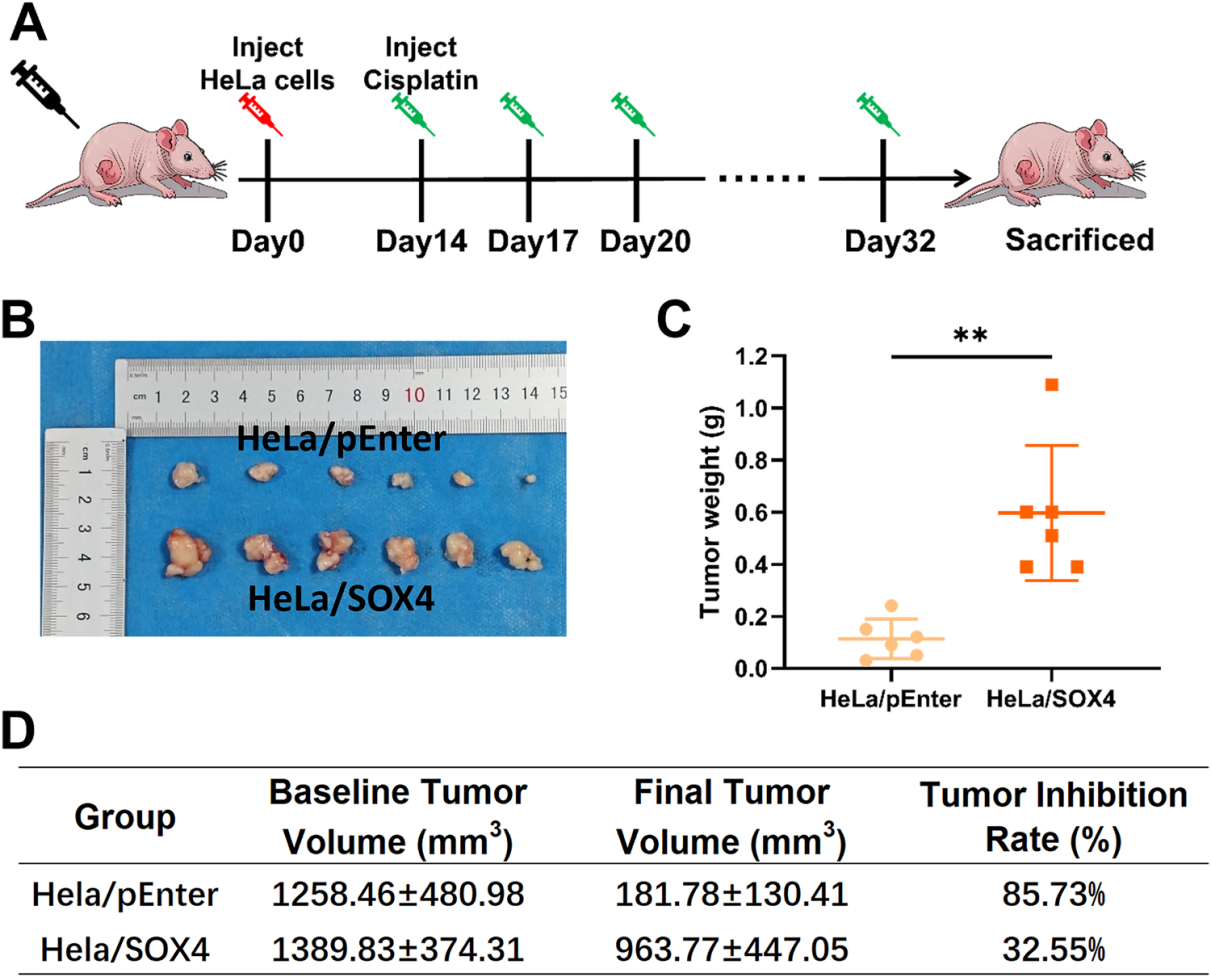
SOX4 promotes cisplatin resistance in cervical cancer cells in vivo. Female BALB/c nude mice (5 weeks old) were subcutaneously injected with HeLa/pEnter or HeLa/SOX4 cells (*n* = 6 per group) at a dose of 2 × 10⁶ cells/200 μL per mouse. When tumors reached approximately 1500 mm³, cisplatin (2 mg/kg) was administered intraperitoneally every 3 days until a significant reduction in tumor size was observed. **A** Schematic diagram showing tumor inoculation and cisplatin treatment in nude mice. **B** Representative images of tumors at the end of the experiment. **C** Tumor weight at the experimental endpoint. **D** Tumor volume monitoring over time, calculating tumor growth inhibition rates. ^*^p < 0.05, ^**^p < 0.01, ^***^p < 0.001.

To further validate whether SOX4-induced cisplatin resistance occurs in vivo, we employed a xenograft model using nude mice. HeLa/pEnter and HeLa/SOX4 cells were subcutaneously injected into separate groups of mice. Once tumor volumes reached 1500 mm³, the mice were treated with cisplatin. When the tumors showed significant reduction and volume differences, the mice were sacrificed, and the tumors were excised to measure their volume and weight. The results indicated that, compared to the HeLa/pEnter group, tumors in the mice injected with HeLa/SOX4 cells were significantly larger in both weight and volume, with the average tumor weight being 5.27 times greater than that of the HeLa/pEnter group. By comparing the tumor volume after each cisplatin injection to the tumor volume prior to the first injection, we calculated the tumor inhibition rate. The results showed that the tumor inhibition rate in the HeLa/SOX4 group was consistently lower than that in the HeLa/pEnter group from the second cisplatin injection to the final injection (Fig. 3A–D). These in vivo findings confirm that SOX4 can induce cisplatin resistance in cervical cancer cells in vivo.

### SOX4 inhibits glycolysis in cervical cancer cells

**Fig. 4:**
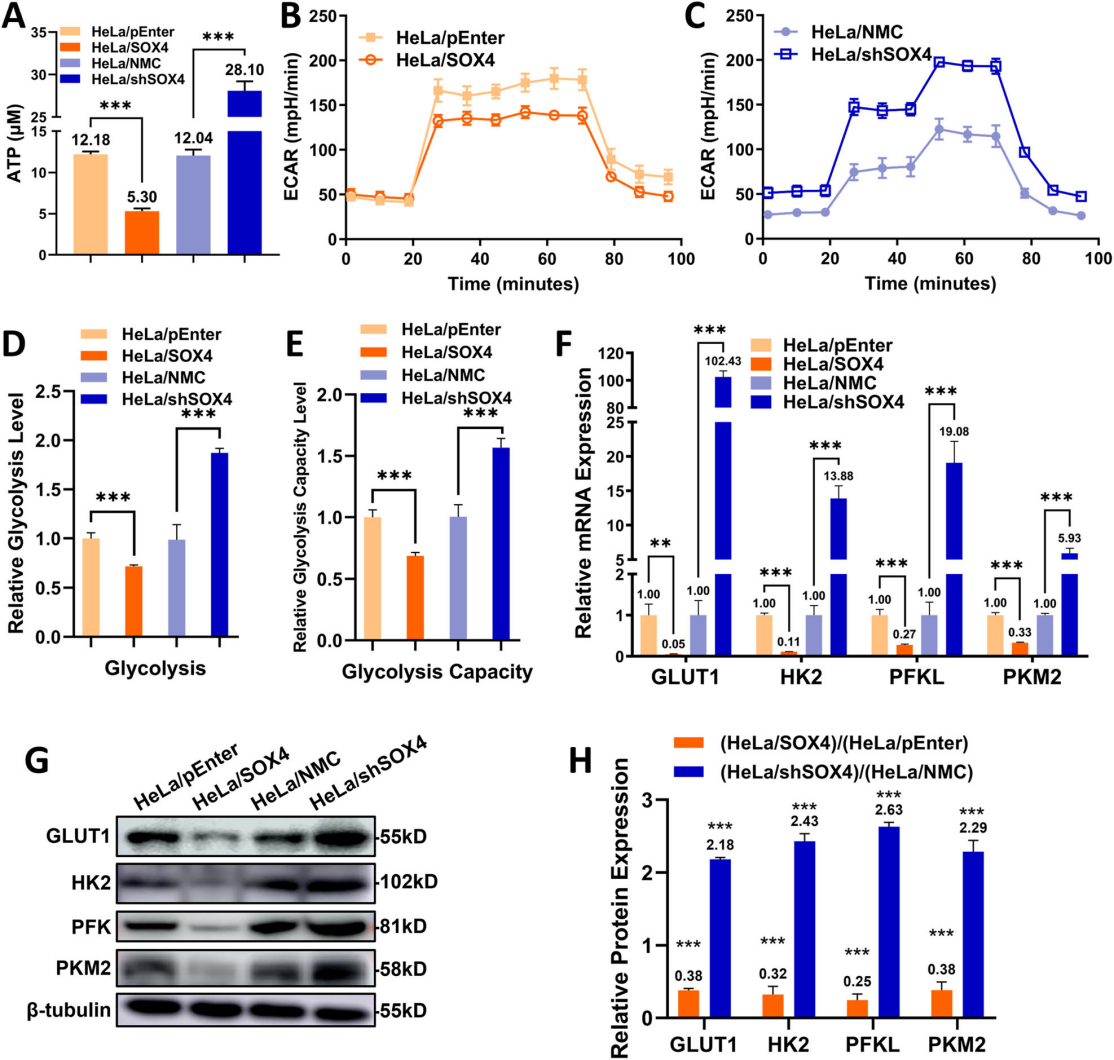
SOX4 inhibits aerobic glycolysis in cervical cancer cells. **A** ATP assay analysis of overall metabolic activity in four SOX4-modified HeLa cell lines. **B**, **C** Measurement of extracellular acidification rate (ECAR) in HeLa/pEnter, HeLa/SOX4, HeLa/NMC, and HeLa/shSOX4 cells (*n* = 5). **D**, **E** Statistical analysis of (**B**, **C**). **F** qPCR analysis of glycolysis-related mRNA expression in SOX4-overexpressing and knockdown HeLa cells. **G** Western blot analysis of glycolysis-related protein expression in SOX4-overexpressing and knockdown HeLa cells. **H** Statistical analysis of **G**. ^*^p < 0.05, ^**^p < 0.01, ^***^p < 0.001.

Emerging evidence has indicated that tumor metabolism plays a crucial role in chemotherapy resistance [29, 30]. Although enhanced glycolysis promotes tumor progression and resistance, some studies show that cisplatin-resistant ovarian cancer cells have reduced glucose uptake [31]. To investigate whether SOX4-induced cisplatin resistance in cervical cancer is related to energy metabolism, we first examined intracellular ATP production. The results showed that the ATP levels in HeLa/SOX4 cells were only 43.5% of those in HeLa/pEnter cells, while HeLa/shSOX4 cells had 2.33 times the ATP levels compared to HeLa/NMC cells (Fig. 4A). Seahorse analysis revealed a decrease in the extracellular acidification rate (ECAR) levels in HeLa/SOX4 cells and an increase in HeLa/shSOX4 cells (Fig. 4B–E). These findings suggest that SOX4 overexpression suppresses glycolytic activity in cervical cancer cells.

qPCR analysis revealed that the mRNA levels of GLUT1, HK2, PFKL, and PKM2 were significantly lower in HeLa/SOX4 cells, reaching only 5.0%, 11.1%, 27.5%, and 32.9%, respectively, compared to HeLa/pEnter cells. Conversely, in HeLa/shSOX4 cells, these four genes were significantly upregulated, with increases of 102.4-fold, 13.9-fold, 19.1-fold, and 5.9-fold relative to HeLa/NMC cells (Fig. 4F). Western blot confirmed corresponding protein changes (Fig. 4G, H).

**Fig. 5:**
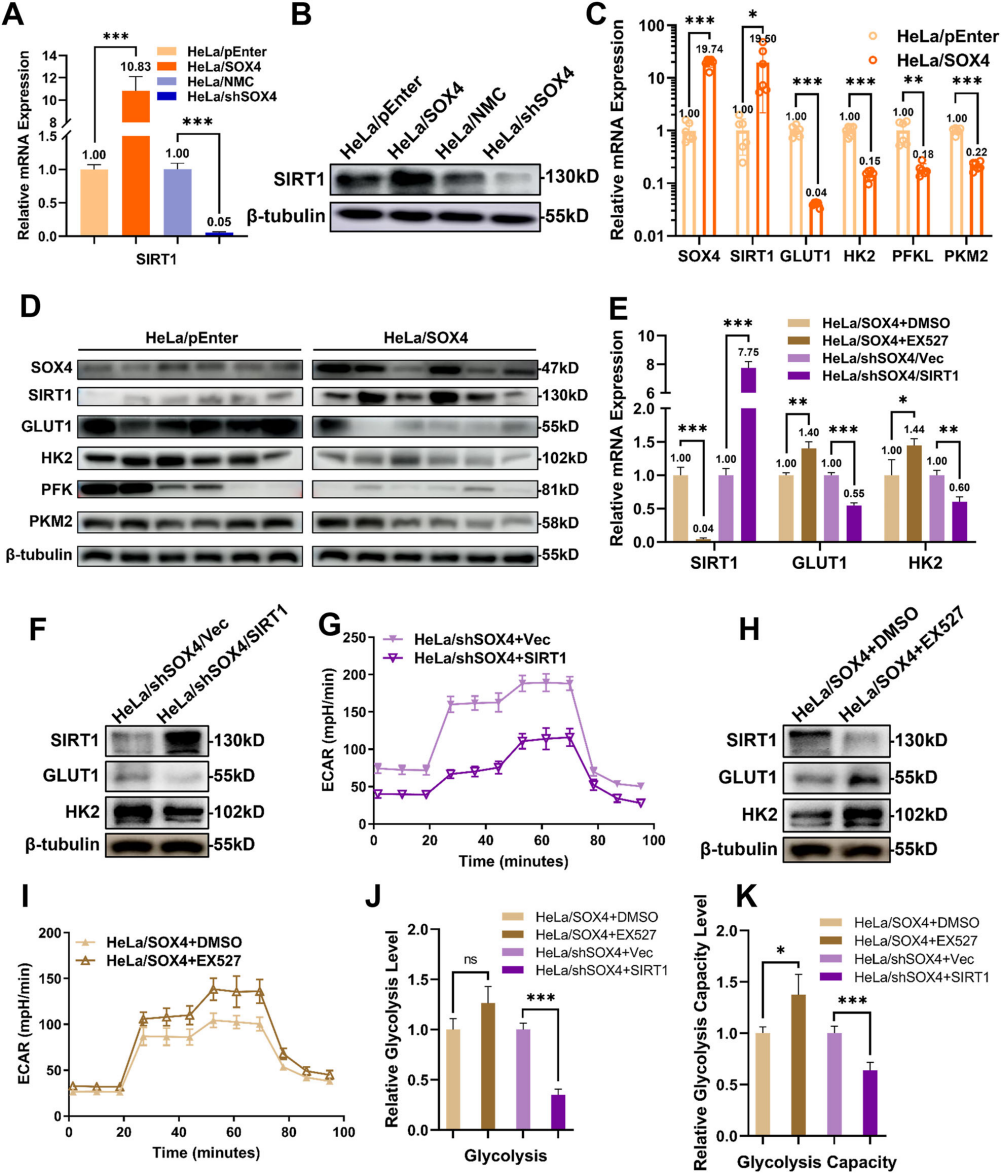
SOX4 regulates glycolysis in cervical cancer cells via SIRT1 upregulation. **A** qPCR analysis of SIRT1 mRNA expression in SOX4-modified HeLa cells. **B** Western blot analysis of SIRT1 protein expression in SOX4-modified HeLa cells. To investigate the effect of SIRT1 modulation, EX527 was added to HeLa/SOX4 cells and SIRT1 was overexpressed in HeLa/shSOX4 cells. **C** qPCR analysis of SOX4, SIRT1, and glycolysis markers in tumor tissues. **D** Western blot analysis of SOX4, SIRT1, and glycolysis markers in tumor tissues. **E** qPCR analysis of SIRT1 and glucose metabolism markers (GLUT1 and HK2) expression. **F**, **H** Western blot analysis of SIRT1 and glucose metabolism markers (GLUT1 and HK2) expression. **G**, **I** Seahorse analysis of glycolysis in cells. **J**, **K** Statistical analysis of (**E**, **G**). ^*^p < 0.05, ^**^p < 0.01, ^***^p < 0.001.

To determine whether SOX4 similarly regulates glycolysis in vivo, we analyzed tumor tissues from nude mice. qPCR and Western blot results revealed that in the HeLa/SOX4 group, SOX4 expression was elevated 19.74-fold, while glycolytic markers GLUT1, HK2, PFK, and PKM2 were decreased to 3.99%, 14.56%, 18.41%, and 21.63% of the levels observed in the HeLa/pEnter group, respectively. Western blot results showed similar trends, consistent with the in vitro findings (Fig. 5C, D). These in vivo results confirm that SOX4 inhibits glycolysis in cervical cancer cells.

In summary, these results demonstrate that SOX4 suppresses glycolysis in cervical cancer. Since cisplatin predominantly targets rapidly proliferating cells, we propose that SOX4 promotes cisplatin resistance by reducing glycolytic activity, thereby enabling cells to evade cisplatin-induced cytotoxicity.

### SOX4 inhibits cervical cancer cell glycolysis via SIRT1 upregulation

SIRT1 has been widely recognized as a key regulator of glycolysis. To investigate whether SIRT1 is involved in SOX4-mediated glycolytic regulation in cervical cancer cells, we evaluated SIRT1 expression in SOX4-modified HeLa cells. qPCR results showed a positive correlation between SIRT1 and SOX4 expression levels. In HeLa/SOX4 cells, SIRT1 mRNA was 10.83 times higher than in HeLa/pEnter cells, whereas it was reduced to 5% of control level in HeLa/shSOX4 cells (Fig. 5A). Western blot analysis corroborated these observations (Fig. 5B). Consistent trends were also observed in tumor tissues from nude mice using both qPCR and Western blot (Fig. 5C, D). These findings support the hypothesis that SOX4 regulates glycolysis in HeLa cells through SIRT1.

To further verify that SOX4 modulates glycolysis via SIRT1, we performed a rescue experiment by overexpressing SIRT1 in HeLa/shSOX4 cells. qPCR results showed that SIRT1 overexpression increased its mRNA level by 7.75-fold, while the expression of glycolysis-related markers GLUT1 and HK2 decreased to 54.7% and 60.5%, respectively, relative to baseline (Fig. 5E). Western blot results were consistent with these findings (Fig. 5F). Seahorse analysis further supported these observations (Fig. 5G, J, K). These results indicate that SOX4 inhibits glycolysis in HeLa cells by upregulating SIRT1.

Conversely, treatment of HeLa/SOX4 cells with the SIRT1 inhibitor EX527 led to a reduction in SIRT1 mRNA to 4.17% of baseline, while the mRNA expression of GLUT1 and HK2 increased by 1.40- and 1.45-fold, respectively (Fig. 5E). Western blot and Seahorse analyzes confirmed these findings (Fig. 5H–K). These results suggest that the inhibition of SIRT1 can reverse SOX4-mediated inhibition of glycolysis.

### SOX4 induces cisplatin resistance in cervical cancer cells via SIRT1

**Fig. 6:**
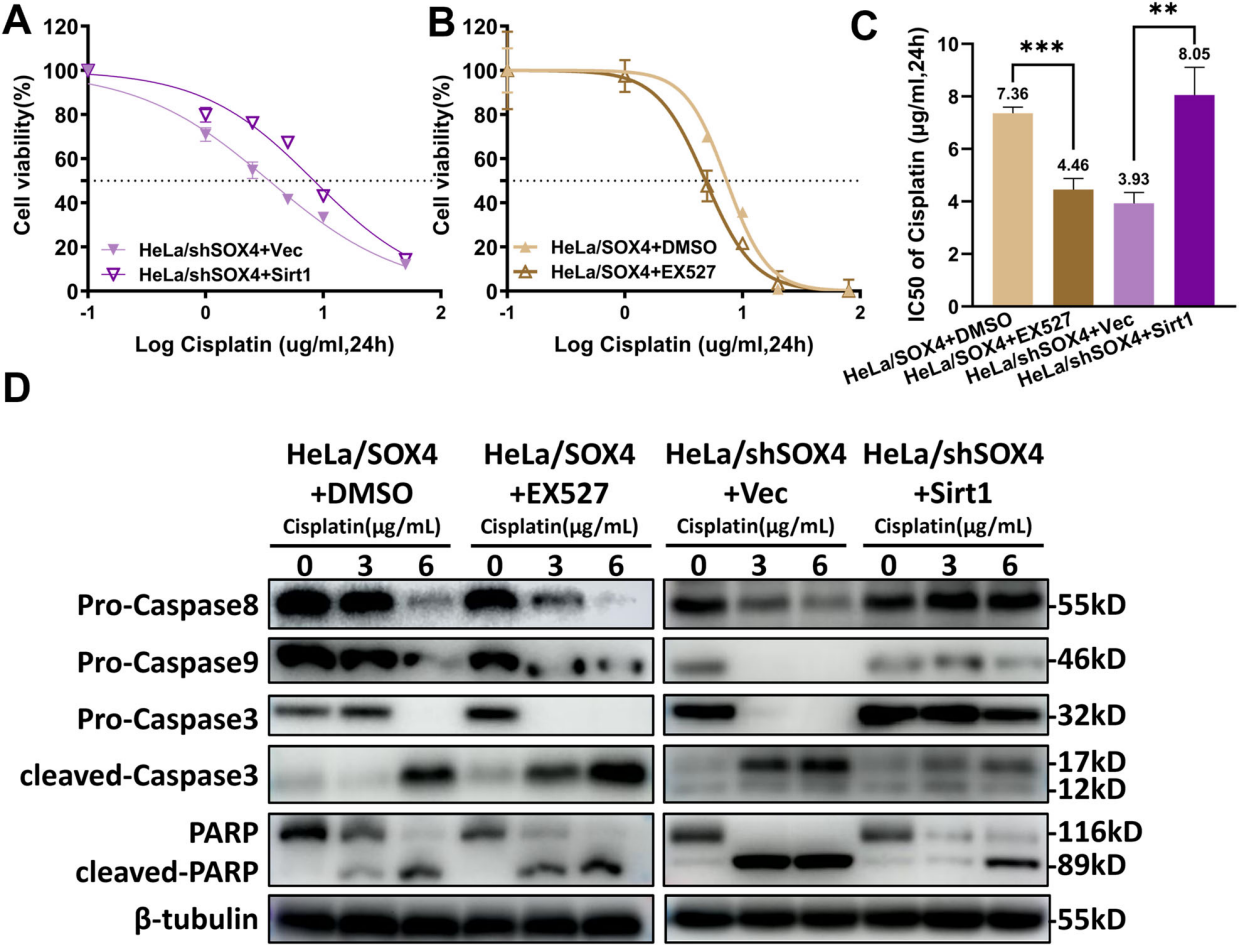
SOX4 induces cisplatin resistance in cervical cancer cells via SIRT1. HeLa/shSOX4 cells were transfected to overexpress SIRT1, and EX527 was added to HeLa/SOX4 cells. Subsequently, the cells were treated with different concentrations of cisplatin. **A**–**C** IC50 values of cisplatin in SIRT1-modified HeLa cells were measured by CCK-8 assay. **D** Western blot analysis of apoptosis-related proteins. ^*^p < 0.05, ^**^p < 0.01, ^***^p < 0.001.

To further verify whether SOX4 modulates apoptosis and drug resistance via the same mechanism, increasing concentrations of cisplatin were added to the HeLa/SOX4 cells with SIRT1 inhibition and the HeLa/shSOX4 cells with SIRT1 overexpression. CCK-8 assay showed that SIRT1 inhibition in HeLa/SOX4 cells reduced the IC50 from 7.36 to 4.46, whereas SIRT1 overexpression in HeLa/shSOX4 cells increased the IC50 from 3.93 to 8.05 (Fig. 6A–C). Western blot analysis revealed that, when the cisplatin concentration was 3 μg/mL, the Caspase3, Caspase8, Caspase9, and PARP proteins in the HeLa/SOX4 cells with DMSO treatment showed no significant activation or had low activation levels. However, HeLa/SOX4 cells treated with EX527 exhibited marked activation of these apoptotic proteins at the same cisplatin concentration. In contrast, control HeLa/shSOX4 cells displayed clear activation of all four apoptotic markers at 3 μg/mL cisplatin, whereas HeLa/shSOX4 cells overexpressing SIRT1 maintained low levels of protein activation even at 6 μg/mL cisplatin. These results suggest that SOX4 promotes cisplatin resistance in HeLa cells through a SIRT1-dependent mechanism (Fig. 6D).

## Discussion

Chemoresistance has long been a major challenge in tumor treatment. It leads to reduced efficacy of conventional chemotherapeutic agents and complicates treatment regimens. The mechanisms underlying resistance are multifactorial, including drug efflux, drug metabolism, DNA repair, and the regulation of intracellular signaling pathways. Cisplatin, a first-line chemotherapeutic drug, demonstrates efficacy in various solid tumors, including cervical cancer. Our previous studies have shown that SOX4 is highly expressed in cervical cancer, and its high expression is positively correlated with poor prognosis. Additionally, SOX4 promotes chemotherapy resistance in cervical cancer cells by regulating ABCG2 [32]. Recent studies have suggested that tumor resistance may also be related to metabolic reprogramming, and an increase in glycolysis levels can contribute to cisplatin resistance in tumor cells [19, 20]. Moreover, HPV infection, which is the primary etiological factor of cervical cancer, may also be associated with platinum resistance. For instance, it has been reported that in HPV-positive cervical cancer cell lines, the transmembrane protein TMEM45A is highly expressed, and its expression is further elevated in cisplatin-resistant cells. Knockdown of TMEM45A by shRNA reduced the cisplatin IC50 and resistance index, and increased apoptosis, thereby reversing cisplatin resistance [33]. These observations imply a potential connection between HPV-associated elements and cisplatin resistance, prompting us to investigate the specific mechanisms through which SOX4 drives this process.

SOX4, as an important developmental transcription factor, plays a crucial role in regulating stem cell characteristics, differentiation, proliferation, migration, and invasion, with substantial research having been conducted in these areas. For instance, SOX4 promotes EMT in tumor cells by interacting with the TGF-β/SMAD pathway [34–36]; in prostate cancer, SOX4 forms a positive feedback loop with the PI3K/AKT pathway, promoting prostate cancer progression [37]; SOX4 can directly bind with β-catenin [38–41], and further studies have shown that in prostate cancer, SOX4 regulates AKT phosphorylation and inhibits GSK3β, facilitating crosstalk between the PI3K/AKT and Wnt/β-catenin pathways [38–41]. Research has also indicated that SOX4 can induce resistance to tamoxifen in breast cancer [42], and in colorectal cancer, it can induce resistance to 5-fluorouracil [43]. Nevertheless, the roles of SOX4 in tumor metabolism, apoptosis, and resistance mechanisms remain inadequately explored. Therefore, this study focuses on elucidating the functions of SOX4 in metabolic reprogramming and chemotherapy resistance in cervical cancer, with the goal of identifying novel therapeutic targets to improve clinical outcomes and overcome treatment resistance.

Cisplatin exerts its anti-tumor effects through multiple mechanisms, with the most prominent being the activation of DNA damage response and induction of mitochondrial apoptosis [44]. Recent studies have suggested that chemoresistance may also be linked to metabolic reprogramming, where increased glycolysis can lead to resistance of tumor cells to platinum-based chemotherapeutic drugs [19, 20]. To explore this mechanism, we performed metabolic assays in SOX4-modulated cervical cancer cells, and the results indicated that SOX4 overexpression significantly downregulated GLUT1 expression, accompanied by reduced intracellular glycolytic levels and ATP production. Whether SOX4 mediates this effect by regulating GLUT1 expression, leading to reduced glucose uptake and thereby reducing glycolytic activity, or whether SOX4 directly regulates the expression of glycolysis-related enzymes remains unclear and requires further investigation.

The regulatory role of SIRT1 in tumor metabolism was introduced earlier. Studies have shown that SIRT1 can promote GLUT1 expression and glycolysis in bladder cancer [45]. In contrast, SIRT1 can bind with FOXO3 and NRF1 in fatty liver, thereby inhibiting glycolysis [46]. These studies highlight the tissue-specific functions of SIRT1. However, the mechanism by which SIRT1 regulates glycolysis in cervical cancer remains unclear. We measured SIRT1 expression in SOX4-modulated HeLa cells and found that SIRT1 expression was positively correlated with SOX4, and SOX4 regulates both glycolytic suppression and cisplatin resistance through SIRT1. Our research demonstrates that SIRT1 inhibits glycolysis in cervical cancer, expanding our understanding of its role. These findings suggest that SIRT1 could be a potential therapeutic target for cervical cancer, particularly in cases with high SOX4 expression.

In conclusion, we have identified a novel mechanism by which SOX4 induces cisplatin resistance in cervical cancer cells. SOX4 upregulates SIRT1, leading to suppression of glycolysis. This metabolic attenuation allows cells to evade cisplatin-induced apoptosis by reducing their metabolic activity, thereby promoting cisplatin resistance. Our results underscore the therapeutic potential of targeting the SOX4/SIRT1 pathway to overcome cisplatin resistance in cervical cancer.

## Materials and methods

### Protein extraction and Western blotting

Cervical cancer cells and tumor tissues from nude mice were lysed in cold RIPA buffer (Beyotime, Shanghai, China) supplemented with protease inhibitors (Targetmol, Boston, USA). Protein concentrations were determined using a BCA Protein Assay Kit (Beyotime, Shanghai, China). After separation by SDS-PAGE, the proteins were transferred onto PVDF membranes (Millipore, Billerica, USA). Detection was performed using specific primary antibodies, followed by HRP-conjugated anti-rabbit or anti-mouse IgG secondary antibodies. (Huabio, Hangzhou, China). Signals were visualized using an enhanced chemiluminescence (ECL) detection kit (Vazyme, Nanjing, China).

### Antibodies and reagents

The primary antibodies used in this study were as follows: SOX4 (1:1000, Invitrogen, PA5-72163), caspase-3 (1:1000, Huabio, ET1608-64), caspase-8 (1:1000, Huabio, PSH04-77), caspase-9 (1:1000, Huabio, R1308-12), PARP1 (1:1000, Proteintech, 13371), GLUT1 (1:1000, Abways, CY1071), HK2 (1:1000, Huabio, HA500186), PFK (1:1000, Abways, CY8047), PKM2 (1:1000, Abways, CY5764), SIRT1 (1:1000, Proteintech, 13161), and β-tublin (1:5000, Affinity, AF7011).

Cisplatin (HY-17394), EX527 (HY-15452), Staurosporine (HY-15141) were purchased from Vazyme (China). Oxaliplatin was obtained from Hengrui (Jiangsu, China), and carboplatin from Qilu Pharmaceutical (Shandong, China).

### RNA extraction and quantitative real-time PCR (RT-qPCR)

Total RNA was extracted from cervical cancer cells and tumor tissues of nude mice using TRIzol reagent (Vazyme, Nanjing, China). 1 μg of total RNA was reverse-transcribed into cDNA using the HiScript II Q RT SuperMix Kit (Vazyme, Nanjing, China). Quantitative real-time PCR was performed using SYBR Green Master Mix (Vazyme, Nanjing, China) on a Q3 Fast Real-Time PCR System (Thermo Fisher Scientific, Waltham, MA, USA), with β-actin as the internal control. The primer sequences (5′-3′, forward and reverse) are listed in Table 1.

**Table 1:** Primer sequences list.

Gene Name	Primer Sequence (5′-3′)
β-actin	F: CATGTACGTTGCTATCCAGGC R: CTCCTTAATGTCACGCACGAT
SOX4	F: CCTGAACCCCAGCTCAAACT R: GATCATCTCGCTCACCTCGG
SIRT1	F: TAGCCTTGTCAGATAAGGAAGGA R: ACAGCTTCACAGTCAACTTTGT
GLUT1	F: TCTGGCATCAACGCTGTCTTC R: CGATACCGGAGCCAATGGT
HK2	F: TTGACCAGGAGATTGACATGGG R: CAACCGCATCAGGACCTCA
PFKL	F: GTACCTGGCGCTGGTATCTG R: CCTCTCACACATGAAGTTCTCC
PKM2	F: ATAACGCCTACATGGAAAAGTGT R: TAAGCCCATCATCCACGTAGA

### Cell lines

Human cervical cancer HeLa cells were maintained in our laboratory and cultured in Dulbecco’s modified Eagle’s medium supplemented with 10% fetal bovine serum (Invigentech, California, USA) and 1% penicillin-streptomycin (Solarbio, Beijing, China). Cells were incubated at 37 °C in a humidified atmosphere containing 5% CO_2_.

### Plasmid transfection

HeLa cells were seeded in 6-well plates and transfected when cell confluence reached ~80%. The next day, PolyFast Transfection Reagent (MedChemExpress, Shanghai, China) was used to transfect SOX4 and SIRT1 plasmids for 24 h. Transfection efficiency was evaluated by Western blotting and qPCR. Gene upregulation or downregulationwas defined as the change in expression level relative to the control group after plasmid transfection.

### Cell viability assay

Cell viability was assessed using the Cell Counting Kit-8 (CCK-8, Targetmol, Boston, USA) according to the manufacturer’s instructions. Treated HeLa cells were seeded at a density of 4000 cells per well in 96-well plates. After 24 or 48 h of treatment with different concentrations of cisplatin or other drugs, 10 μL of CCK-8 solution was added, and the cells were incubated at 37 °C for 1 h. Absorbance at 450 nm was measured, which is proportional to the number of viable cells in each well.

### Detection of ATP Levels

ATP levels in tumor tissues were measured using an ATP assay kit (Beyotime, Shanghai, China). Samples were lysed with ATP lysis buffer and quantified by BCA protein Assay Kit (Beyotime, Shanghai, China). The reaction mixture, consisting 100 μL of sample or standard and 100 μL of ATP detection solution, was incubated for 3–5 min at room temperature. ATP levels were measured using a microplate reader equipped with a luminometer function and expressed as μmol/L.

### Seahorse assays

The ECAR of HeLa cells was determined using a Seahorse XFe24 Extracellular Flux Analyzer (Agilent, Santa Clara, CA, USA). Cells (1 × 10⁵per well) were seeded into Seahorse XFe24 plates 24 h before the assay. After sequential injections of glucose (100 mM), oligomycin (10 μM), and 2-deoxyglucose (2DG, 1 M), ECAR was measured to assess glycolytic capacity.

### Animals

Five-week-old female nude mice were purchased from Beijing Charles River Laboratory Animal Technology Co., Ltd (Beijing, China). Mice were housed under standard laboratory conditions (23 ± 2 °C, 50% humidity, 12-h light/dark cycle) with free access to food and water. After 1 week of acclimatization, the experiments were conducted. Tumor volume was calculated using the formula: volume = length × width². The tumor inhibition rate was as follows: inhibition rate = (initial tumor volume−final tumor volume)/initial tumor volume. The mean inhibition rate was calculated based on the values of the last 6 mice in each group. All animal procedures were conducted in accordance with the Public Health Service Guide and approved by the Animal Ethics Committee of Henan Medical University (Approval No. XYLL-20240208, Xinxiang, China).

### Statistical analysis

Data are presented as the mean ± SD. All experiments were performed in triplicate in at least three independent repeats. Statistical significance was analyzed using Student’s *t*-test with GraphPad Prism 8.0 software (La Jolla, CA, USA). Statistical significance was defined as ^*^p < 0.05, ^**^p < 0.01, or ^***^p < 0.001.

## Supplementary information

Original western blots

## Data Availability

All data generated or analysed during this study are included in this published article and its supplementary information files.
