# Supplementary material for: SOX4 induces cisplatin resistance in cervical cancer cells by inhibiting aerobic glycolysis
Source: Cell Death Discov. 2026 Mar 14;12:263. doi: 10.1038/s41420-026-02954-x (PMC13230878; doi:10.1038/s41420-026-02954-x)
Supplement: Supplementary file 1 — Original western blots [file 41420_2026_2954_MOESM1_ESM.pdf]

Fig1B

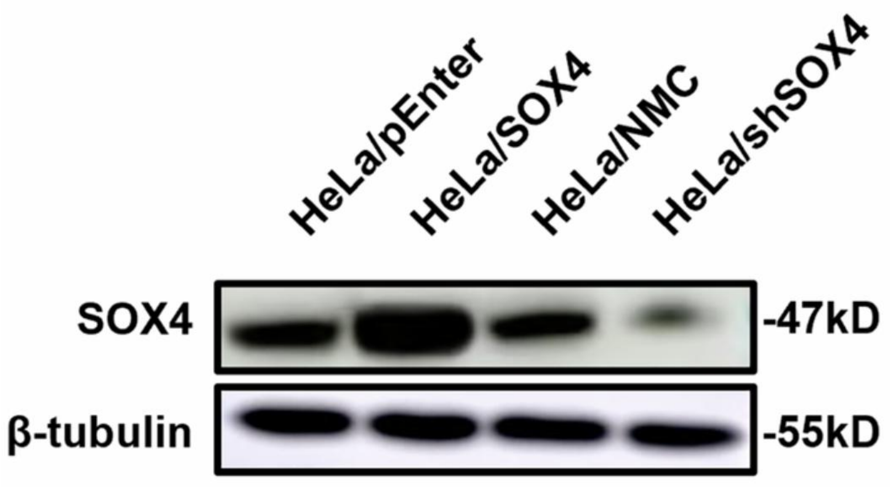

SOX4

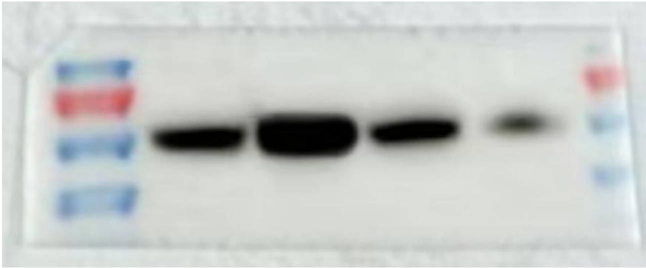

β-tubulin

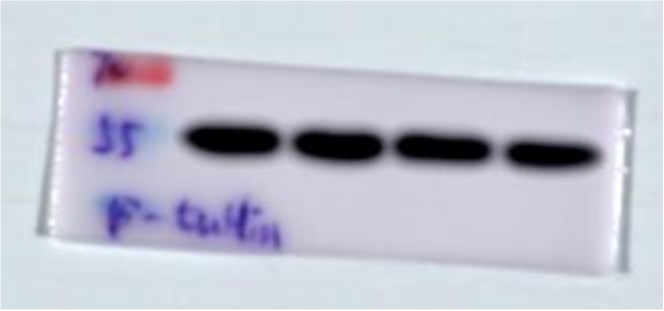

Fig2D

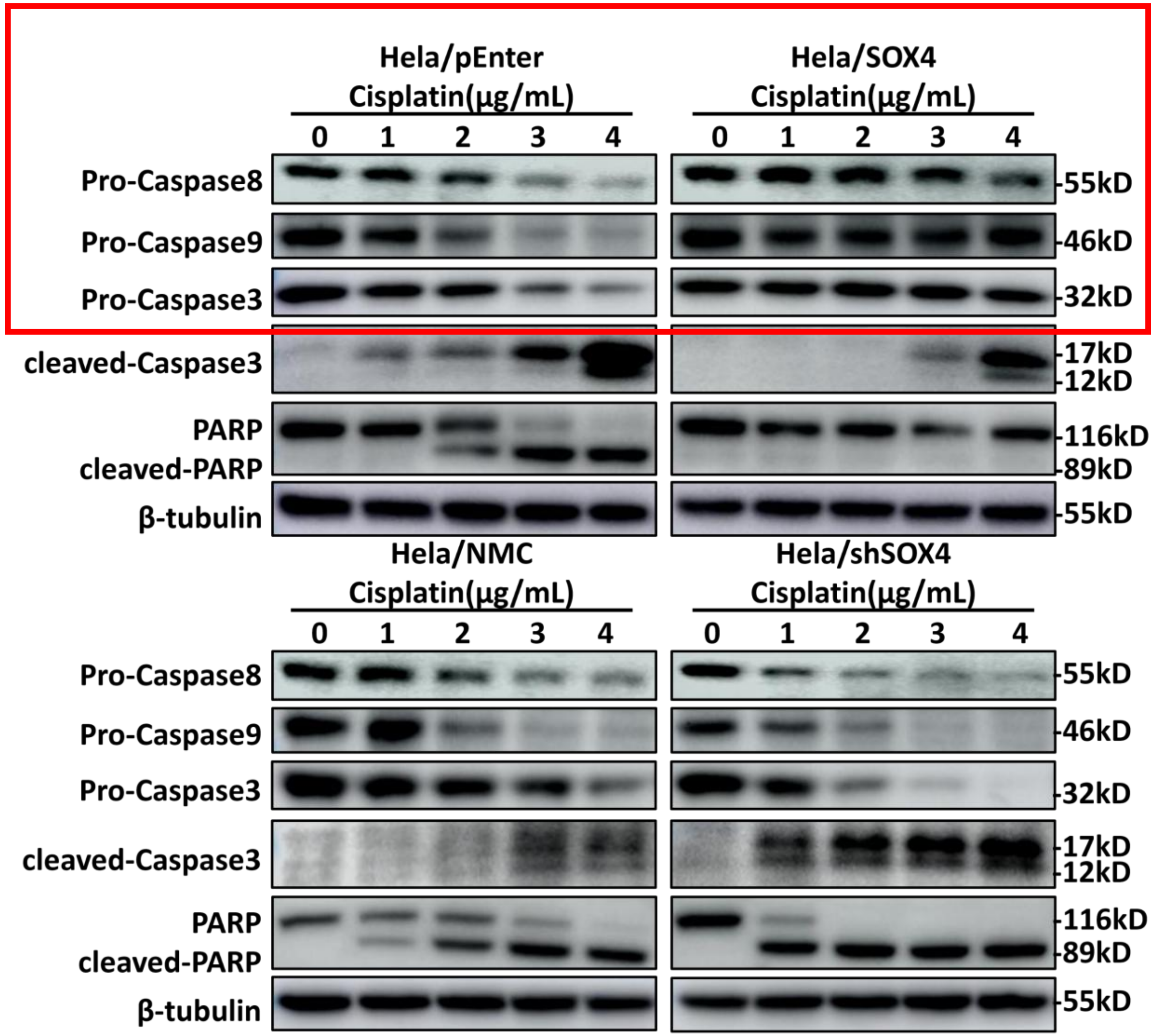
Hela/pEnter
Cisplatin(μg/mL)
0 1 2 3 4
Hela/SOX4
Cisplatin(μg/mL)
0 1 2 3 4
Pro-Caspase8
Pro-Caspase9
Pro-Caspase3
cleaved-Caspase3
PARP
cleaved-PARP
β-tubulin
55kD
46kD
32kD
17kD
12kD
116kD
89kD
55kD
Hela/NMC
Cisplatin(μg/mL)
0 1 2 3 4
Hela/shSOX4
Cisplatin(μg/mL)
0 1 2 3 4
Pro-Caspase8
Pro-Caspase9
Pro-Caspase3
cleaved-Caspase3
PARP
cleaved-PARP
β-tubulin
55kD
46kD
32kD
17kD
12kD
116kD
89kD
55kD

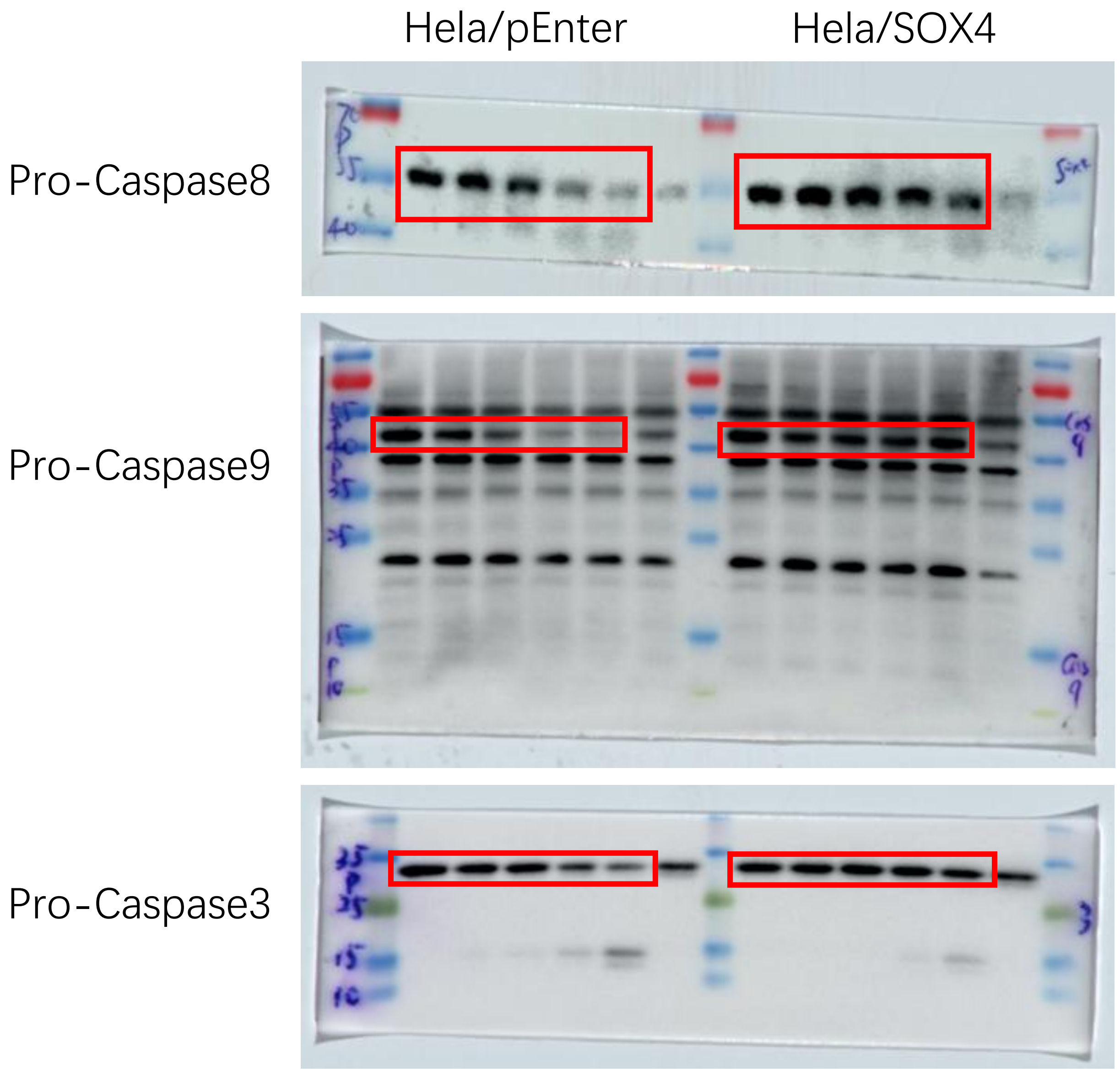
Hela/pEnter
Hela/SOX4
Pro-Caspase8
Pro-Caspase9
Pro-Caspase3

Fig2D

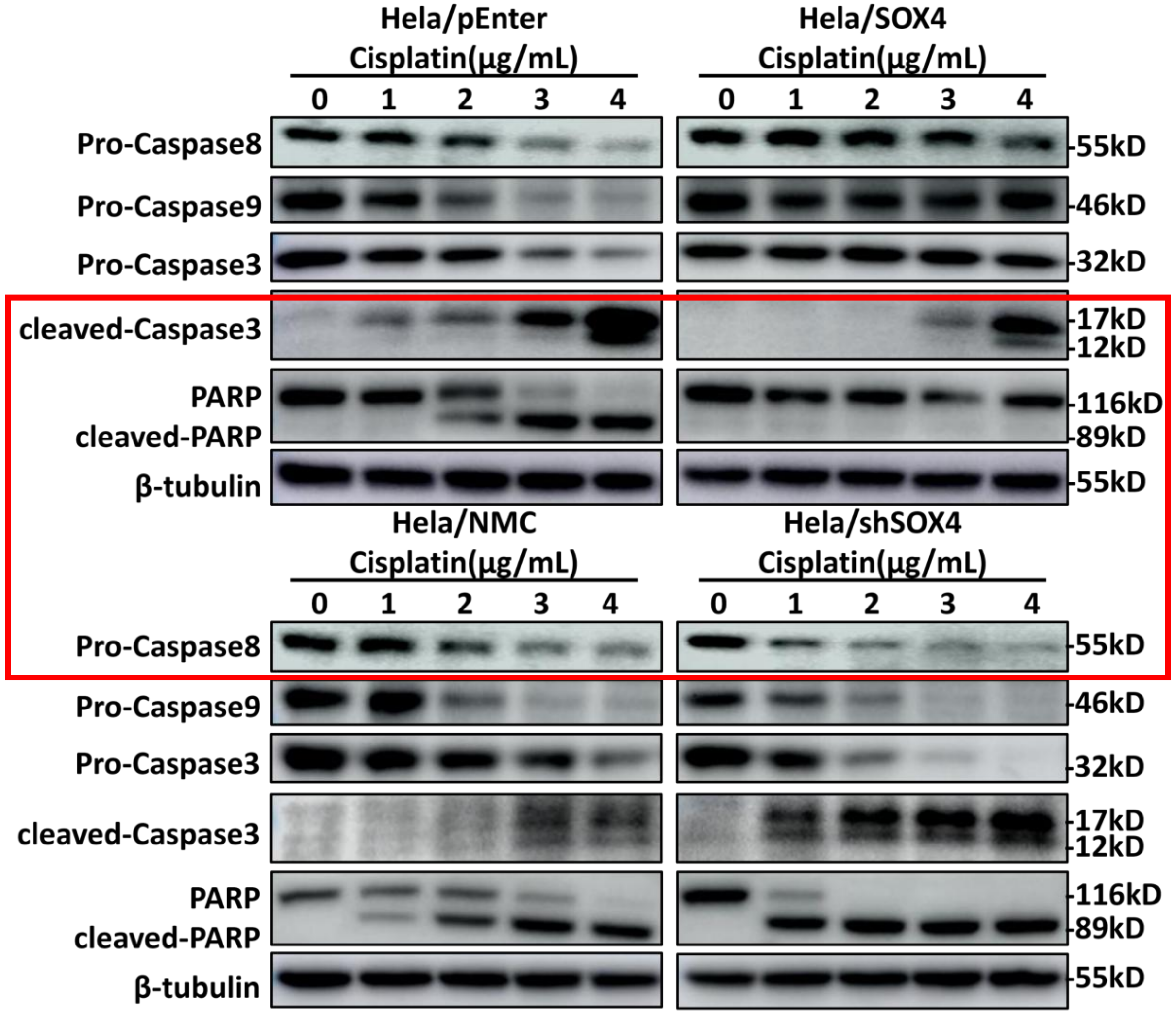
Hela/pEnter
Cisplatin(μg/mL)
0 1 2 3 4
Hela/SOX4
Cisplatin(μg/mL)
0 1 2 3 4
Pro-Caspase8
Pro-Caspase9
Pro-Caspase3
cleaved-Caspase3
PARP
cleaved-PARP
β-tubulin
55kD
46kD
32kD
17kD
12kD
116kD
89kD
55kD
Hela/NMC
Cisplatin(μg/mL)
0 1 2 3 4
Hela/shSOX4
Cisplatin(μg/mL)
0 1 2 3 4
Pro-Caspase8
Pro-Caspase9
Pro-Caspase3
cleaved-Caspase3
PARP
cleaved-PARP
β-tubulin
55kD
46kD
32kD
17kD
12kD
116kD
89kD
55kD

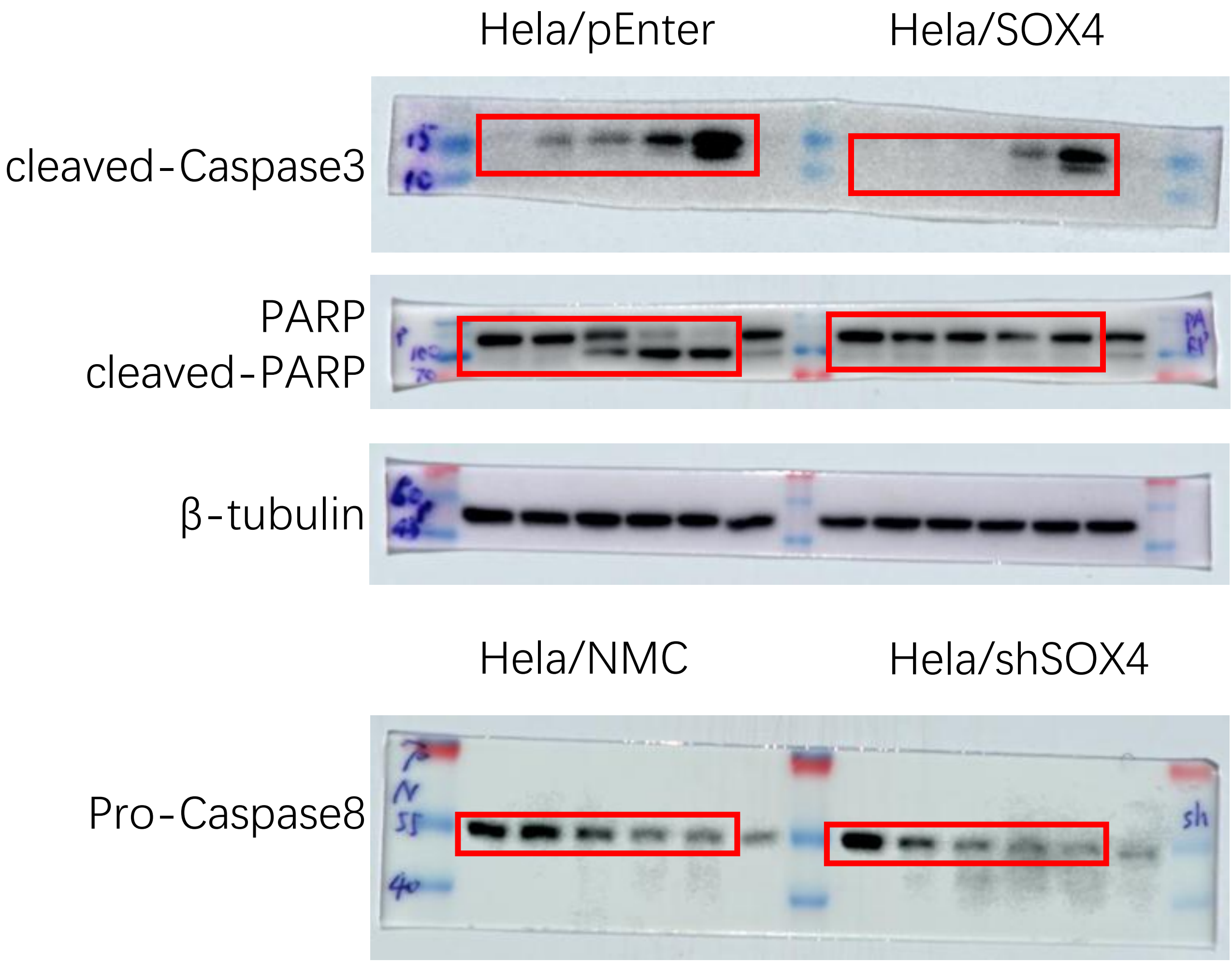
Hela/pEnter
Hela/SOX4
cleaved-Caspase3
PARP
cleaved-PARP
β-tubulin
Hela/NMC
Hela/shSOX4
Pro-Caspase8

Fig2D

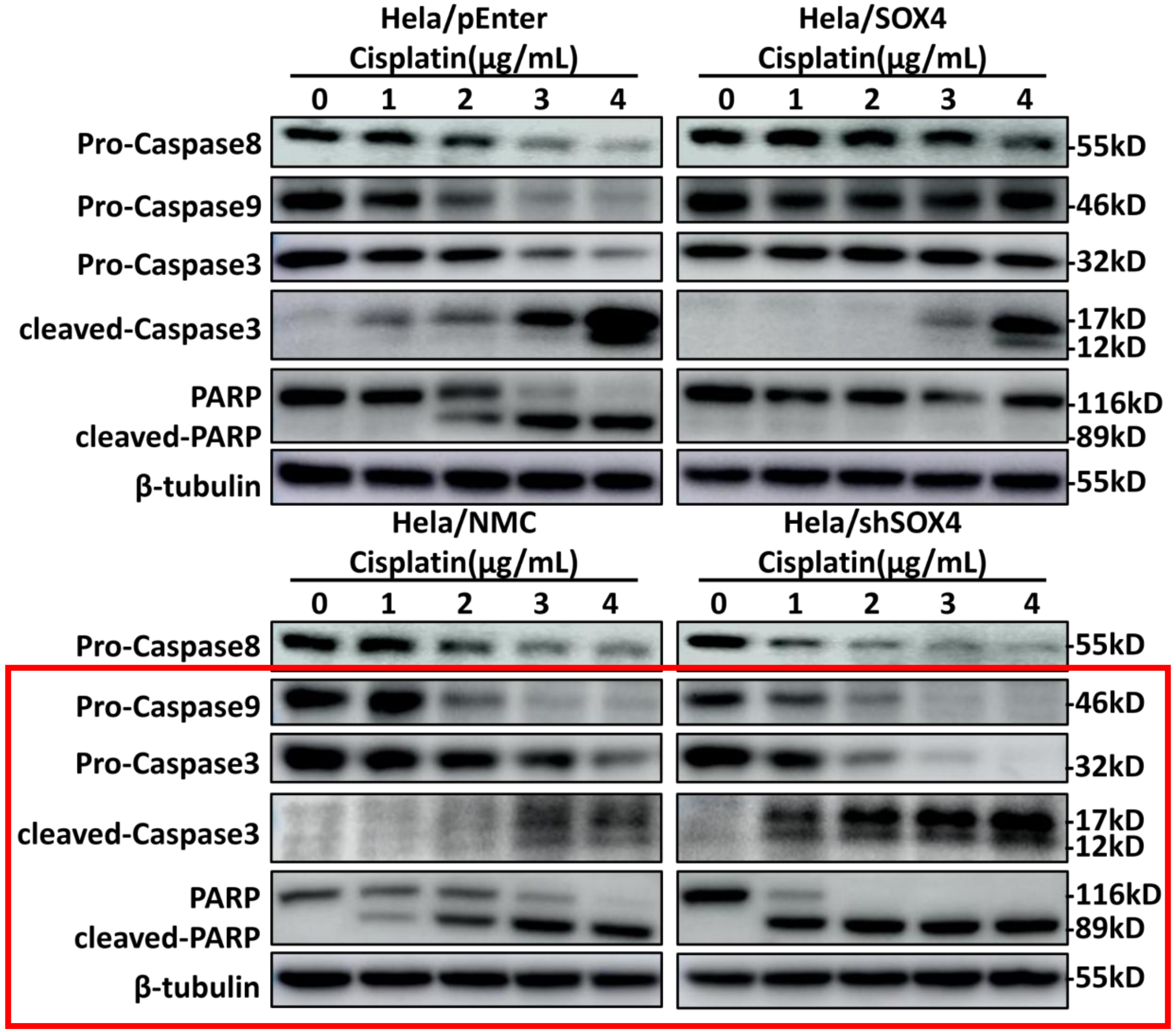
Hela/pEnter
Cisplatin(μg/mL)
0 1 2 3 4
Hela/SOX4
Cisplatin(μg/mL)
0 1 2 3 4
Pro-Caspase8
Pro-Caspase9
Pro-Caspase3
cleaved-Caspase3
PARP
cleaved-PARP
β-tubulin
55kD
46kD
32kD
17kD
12kD
116kD
89kD
55kD
Hela/NMC
Cisplatin(μg/mL)
0 1 2 3 4
Hela/shSOX4
Cisplatin(μg/mL)
0 1 2 3 4
Pro-Caspase8
Pro-Caspase9
Pro-Caspase3
cleaved-Caspase3
PARP
cleaved-PARP
β-tubulin
55kD
46kD
32kD
17kD
12kD
116kD
89kD
55kD

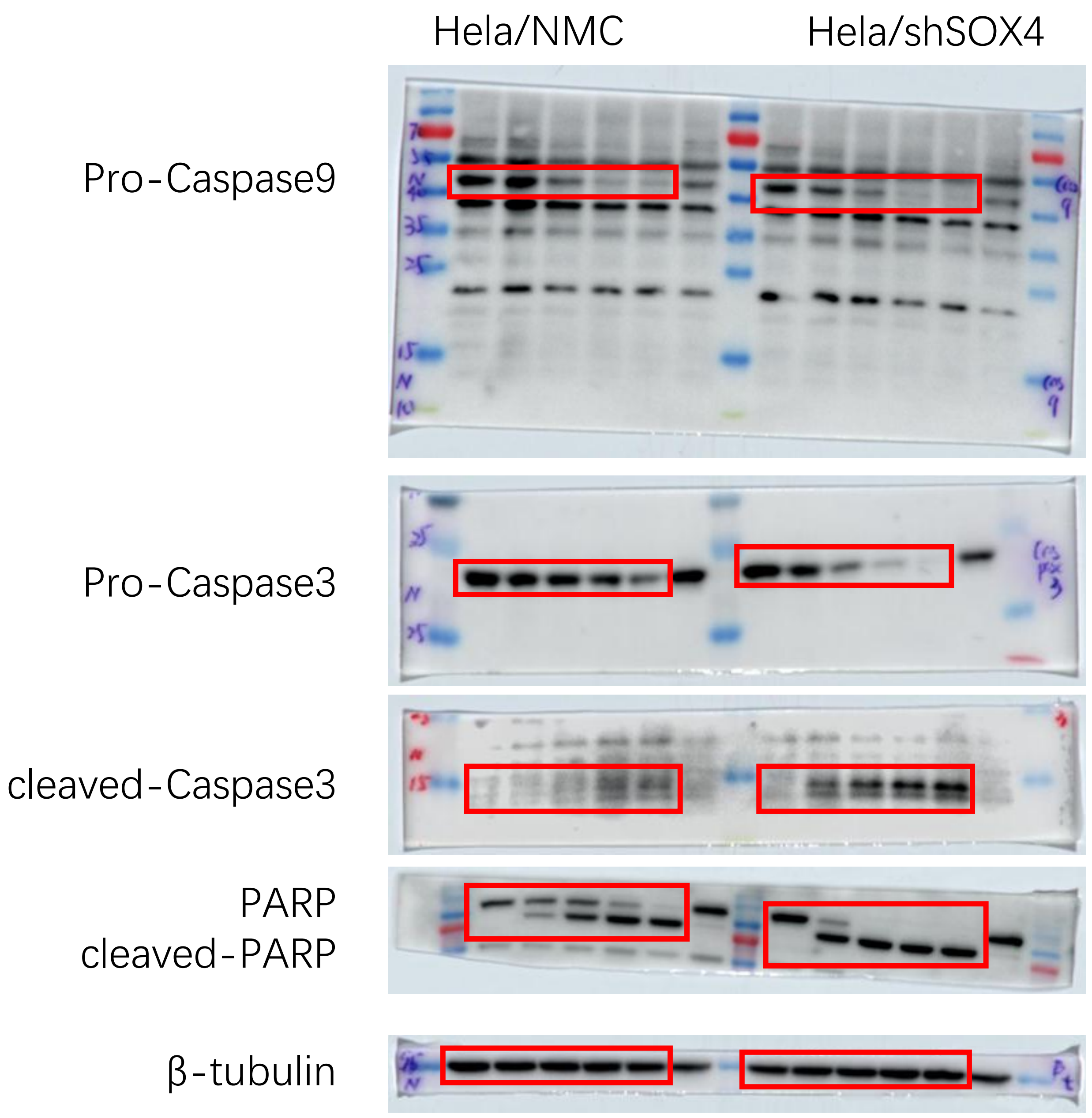
Hela/NMC
Hela/shSOX4
Pro-Caspase9
Pro-Caspase3
cleaved-Caspase3
PARP
cleaved-PARP
β-tubulin

Fig4G

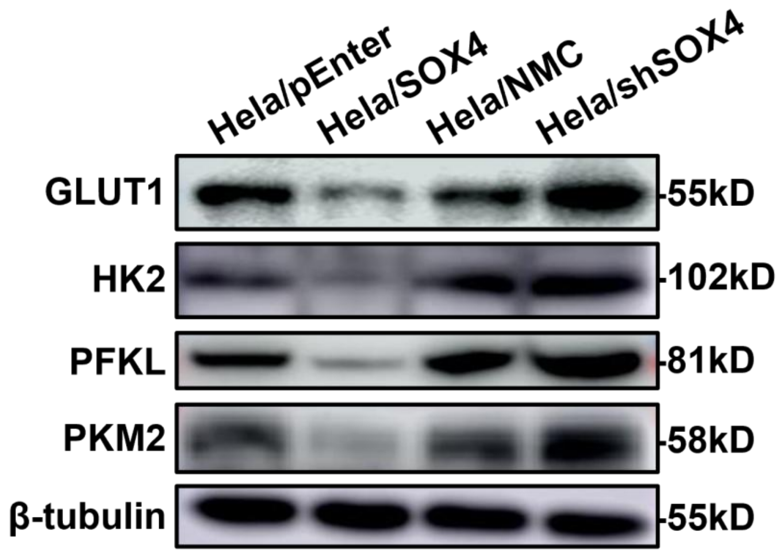
Hela/pEnter
Hela/SOX4
Hela/NMC
Hela/shSOX4
GLUT1
55kD
HK2
102kD
PFKL
81kD
PKM2
58kD
β-tubulin
55kD

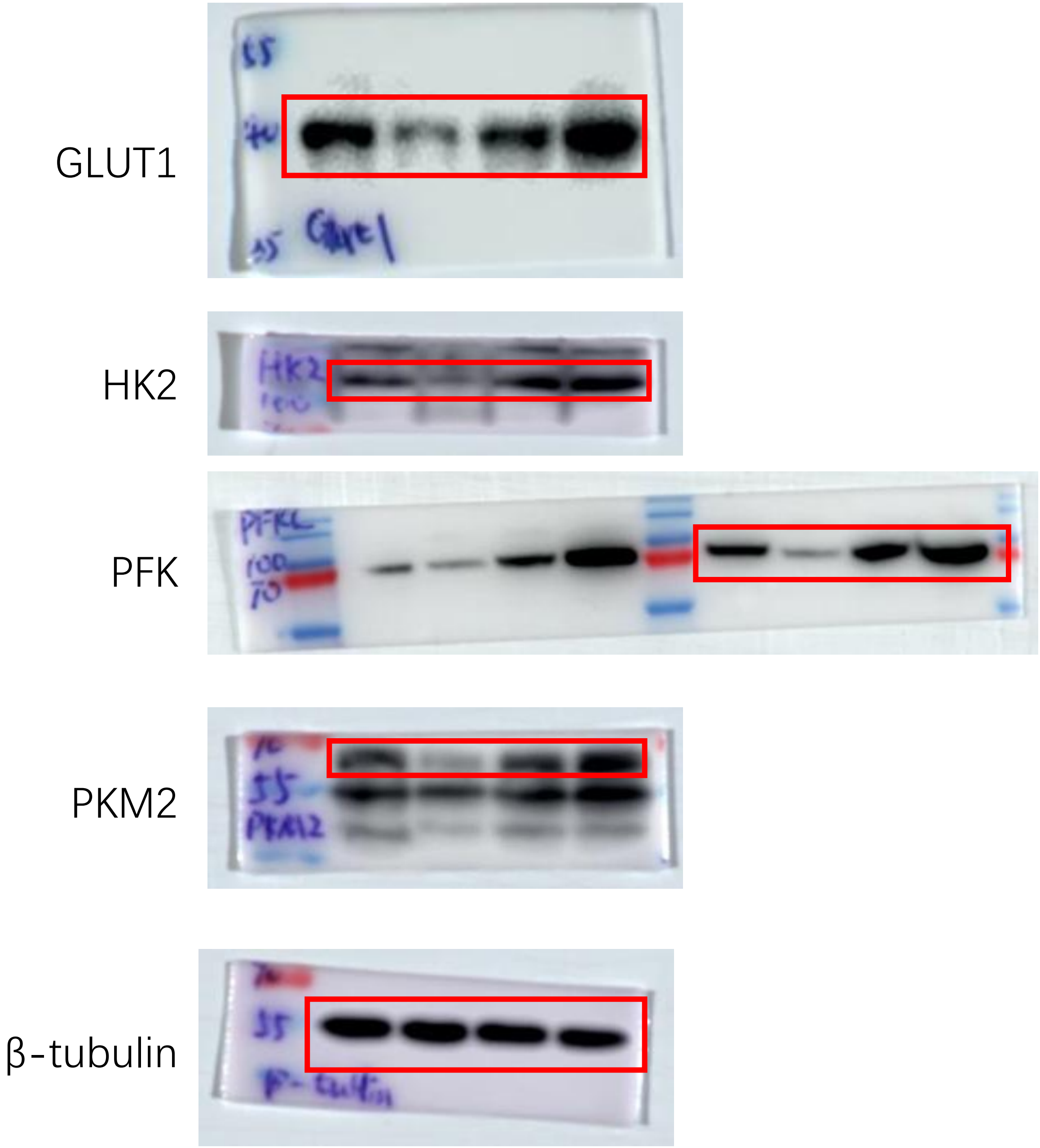
GLUT1
HK2
PFK
PKM2
β-tubulin

Fig5B

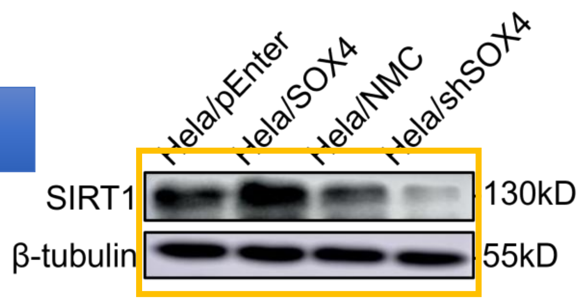

Fig5F

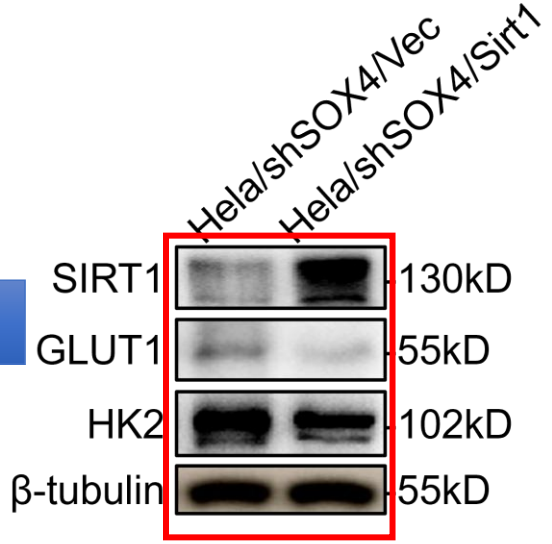

Fig5H

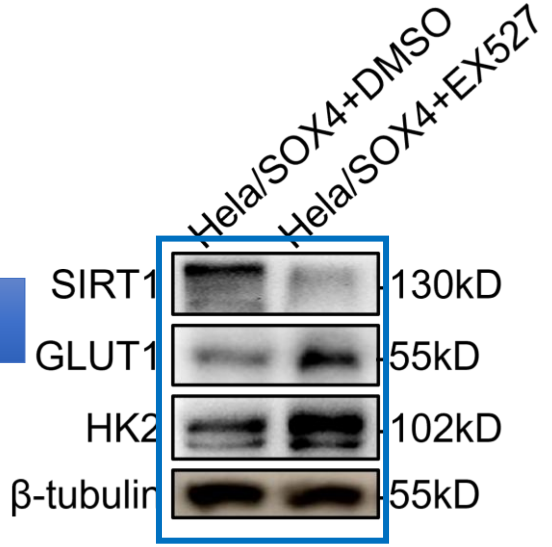

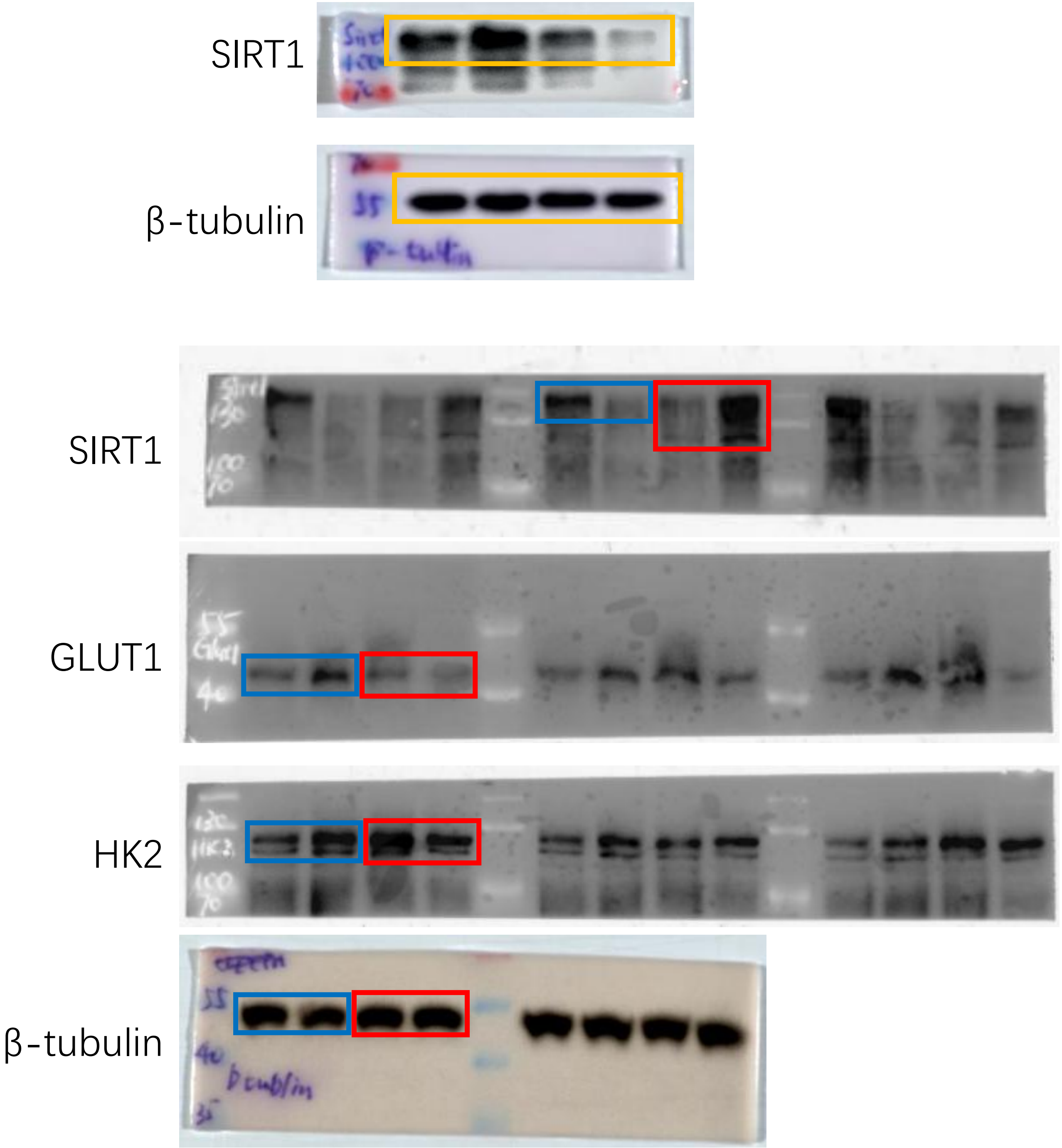

Fig5D

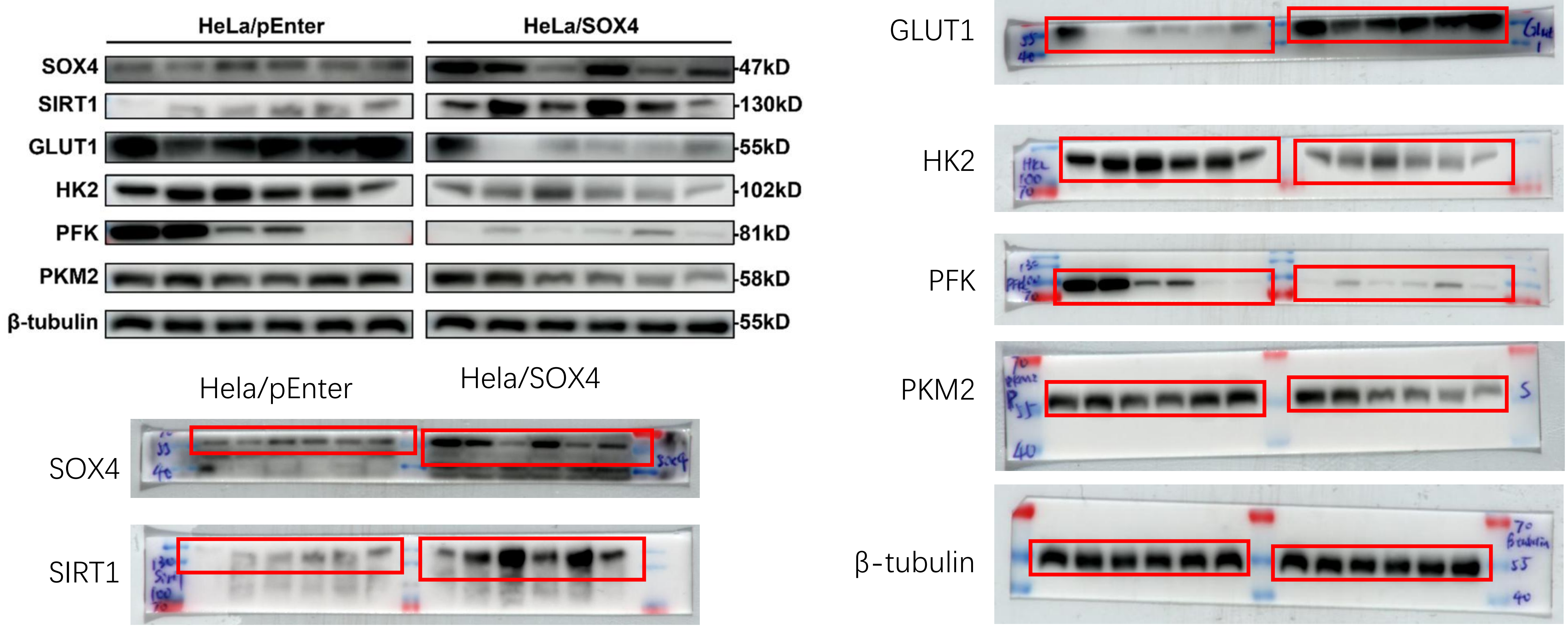
HeLa/pEnter
HeLa/SOX4
SOX4
SIRT1
GLUT1
HK2
PFK
PKM2
β-tubulin
47kD
130kD
55kD
102kD
81kD
58kD
55kD
Hela/pEnter
Hela/SOX4
SOX4
SIRT1
Hela/pEnter
Hela/SOX4
GLUT1
HK2
PFK
PKM2
β-tubulin

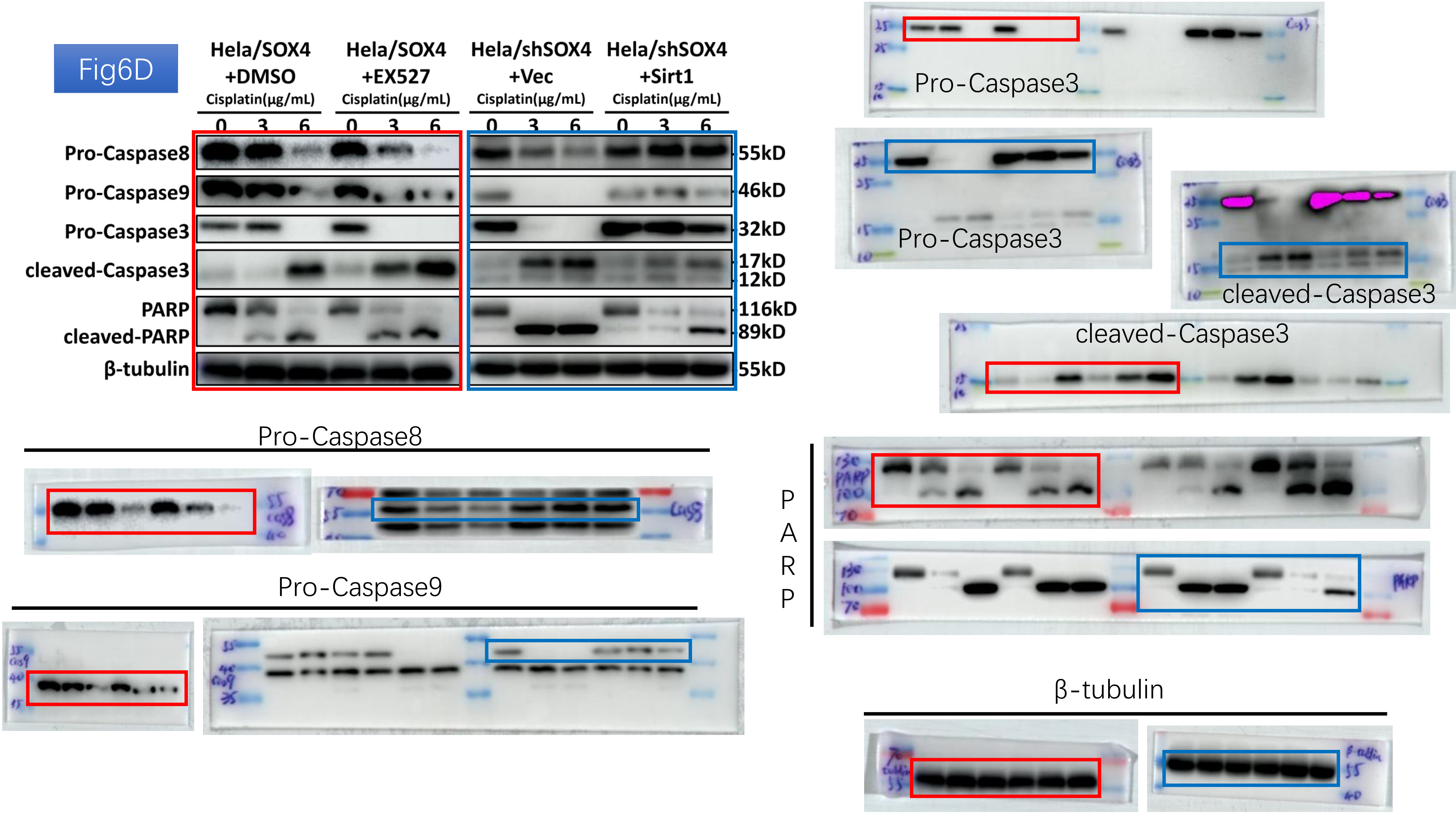
Fig6D
Hela/SOX4 +DMSO
Cisplatin(μg/mL)
Hela/SOX4 +EX527
Cisplatin(μg/mL)
Hela/shSOX4 +Vec
Cisplatin(μg/mL)
Hela/shSOX4 +Sirt1
Cisplatin(μg/mL)
0 3 6 0 3 6 0 3 6 0 3 6
Pro-Caspase8
Pro-Caspase9
Pro-Caspase3
cleaved-Caspase3
PARP
cleaved-PARP
β-tubulin
55kD
46kD
32kD
17kD
12kD
116kD
89kD
55kD
Pro-Caspase3
Pro-Caspase3
cleaved-Caspase3
cleaved-Caspase3
Pro-Caspase8
Pro-Caspase9
PARP
β-tubulin
